# Phase I First-in-Human Study of TRK-950, an IgG1 Antibody Specific to CAPRIN-1, in Patients with Advanced Solid Tumors

**DOI:** 10.1158/2767-9764.CRC-25-0123

**Published:** 2025-07-11

**Authors:** Philippe A. Cassier, Mitesh J. Borad, Sunil Sharma, Bertrand Dubois, Christophe Caux, Fumiyoshi Okano, Daniel D. Von Hoff, Jean-Yves Blay

**Affiliations:** 1 Department of Medical Oncology, Centre Léon Bérard & Centre de Recherche en Cancérologie de Lyon (CRCL), Lyon, France.; 2 Department of Hematology-Oncology, Mayo Clinic Arizona, Mayo Clinic Comprehensive Cancer Center, Phoenix, Arizona.; 3 HonorHealth Research Institute, Scottsdale, Arizona.; 4 Lyon Immunotherapy for Cancer Laboratory, Centre de Recherche en Cancérologie de Lyon (CRCL) INSERM U1052-CNRS UMR5286, Centre Léon Bérard, Université Claude Bernard Lyon 1, Lyon, France.; 5 Toray Industries, Inc., New Frontiers Research Laboratories, Kamakura, Japan.; 6 Translational Genomics Research Institute (TGen) A Part of City of Hope, Phoenix, Arizona.; 7 Department of Medical Oncology, Centre Léon Bérard & Centre de Recherche en Cancérologie de Lyon (CRCL) & Université Claude Bernard Lyon 1, Lyon, France.

## Abstract

**Purpose:**

TRK-950 is a first-in-class humanized antibody targeting cytoplasmic activation/proliferation-associated protein-1, which is strongly expressed on the cell membrane surface in or on most solid tumors but not in or on normal tissues. This first-in-human study investigated the safety profile, pharmacokinetics (PK), and preliminary antitumor activity.

**Patients and Methods:**

Patients with treatment-refractory, locally advanced, or metastatic solid tumors were enrolled in a dose escalation/expansion study. TRK-950 was administered intravenously weekly for 3 weeks in a 28-day cycle, with doses ranging from 3 to 30 mg/kg. Dose expansion included 10 mg/kg weekly and 30 mg/kg biweekly for colorectal cancer and 10 mg/kg weekly for cholangiocarcinoma. The primary objective of this study was to determine its safety, tolerability, and maximum tolerated dose. The secondary objectives were PK, preliminary antitumor activity, and identification of potential biomarkers.

**Results:**

Thirty-six patients received at least one dose of TRK-950. In the dose escalation cohort, the maximum tolerated dose was not reached, and no dose-limiting toxicities were observed up to 30 mg/kg. Common adverse events included abdominal pain, fatigue, constipation, back pain, nausea, and decreased appetite. TRK-950 exhibited a PK profile similar to that of other IgG subclass 1 therapeutic antibodies, with linear PK parameters over the 3 to 30 mg/kg dose range. The best response was stable disease. Notably, one patient with cholangiocarcinoma showed signs of cavitation after approximately 8 months, suggesting potential antitumor activity.

**Conclusions:**

TRK-950 is safe and well tolerated, has a favorable PK profile, and should be further investigated as a monotherapy and in combination with standard treatment for various types of solid tumors.

**Significance:**

TRK-950, a humanized antibody targeting CAPRIN-1, demonstrated good tolerability, no dose-limiting toxicities, a favorable PK profile, and potential antitumor activity in this first-in-human study. Currently, TRK-950 is undergoing Phase Ib and II trials for various cancers, showing promising development potential.

## Introduction

Cytoplasmic activation/proliferation-associated protein-1 (CAPRIN-1) is a highly conserved cytoplasmic phosphoprotein associated with cell proliferation in vertebrates (1, 2). CAPRIN-1 expression decreases when cells cease to divide and increases in all phases of the cell cycle. The lack of CAPRIN-1 in cells can delay progression from the G_1_ phase to the S-phase of the cell cycle (2). Previous reports have shown that CAPRIN-1 can selectively bind to mRNA encoding c-Myc and cyclin D2, which are involved in cell proliferation, and can also directly bind to Ras GTPase–activating protein-binding protein 1 to promote mammalian stress particle formation. Overexpression of CAPRIN-1 can lead to an overall inhibition of stress granule formation in cells (3–11). Several studies have also demonstrated that the intracellular expression of CAPRIN-1 is positively correlated with cancer progression and poor prognosis, suggesting that CAPRIN-1 could be a diagnostic biomarker and a suitable cancer therapeutic target (12–19).

We previously demonstrated that CAPRIN-1 is strongly expressed on the cell membrane surface in or on most solid cancers but not in or on normal tissues and that CAPRIN-1 is present in the cytoplasm in both cancer and normal tissues, which is consistent with previous reports. In cancer cells, part of the total CAPRIN-1 is exposed on the cell membrane surface, a phenomenon that is not observed in noncancerous cells. In addition, we found that cancer stem cells derived from patients with pancreatic cancer also strongly expressed CAPRIN-1 on their cell surface and exhibited enhanced *in vitro* colony-forming activity and *in vivo* tumorigenicity. Based on these prospective data, we concluded that CAPRIN-1 is a promising target for antibody drugs in solid cancers (20).

TRK-950 is a humanized monoclonal IgG subclass 1 (IgG1) antibody specific to human CAPRIN-1 that is under development for the treatment of patients with CAPRIN-1–expressing solid malignant tumors. TRK-950 binds to human CAPRIN-1 with high affinity and can strongly induce tumor cell death through fragment crystallizable-mediated effector functions, including antibody-dependent cell-mediated cytotoxicity and antibody-dependent cell-mediated phagocytosis (ADCP). Consistent with the antibody-dependent antitumor activity, TRK-950 significantly inhibited tumor growth in mouse xenograft models transplanted with several human cancer cells. Furthermore, a comprehensive series of toxicology studies demonstrated that TRK-950 had a favorable safety profile in cynomolgus monkeys at doses up to 200 mg/kg. The cynomolgus monkeys’s no-observed-adverse-effect level of 200 mg/kg provided a predicted safety margin 67-fold above the clinical starting dose in the initial clinical trial (20).

Based on these promising results in nonclinical studies, we conducted a phase I study to establish a safe dose, determine the pharmacokinetics (PK), and document the preliminary antitumor activity of TRK-950 given intravenously in patients with progressive, locally advanced, or metastatic solid tumors.

## Patients and Methods

### Study design and objectives

This first-in-human phase I study (NCT02990481) was an open-label, multicenter, dose-escalation, nonrandomized study of TRK-950 in patients with progressive locally advanced or metastatic solid tumors. The primary objective of this study was to determine the safety, tolerability, and maximum tolerated dose (MTD) of TRK-950. The secondary objectives were to determine the PK of TRK-950 and observe the preliminary antitumor activity of TRK-950. The exploratory objectives were to identify potential biomarkers that may help predict the response to TRK-950 and to establish the dose of TRK-950 recommended for future phase II studies.

For the dose escalation cohort, a traditional 3 + 3 design was used to evaluate dose-limiting toxicity (DLT) and to identify the MTD. The starting dose was 3 mg/kg administered intravenously over 60 minutes weekly for 3 weeks, followed by a 1-week rest period. Depending on the safety findings, dose escalations could proceed up to 30 mg/kg weekly for 3 weeks, followed by a 1-week rest period. Patients were administered TRK-950 intravenously on days 1, 8, and 15 of a 28-day cycle in the dose escalation cohort. After enrollment in the dose escalation was completed, 14 additional patients who were enrolled in the colorectal cancer cohort were administered TRK-950 intravenously weekly (10 mg/kg on days 1, 8, 15, and 22) or biweekly (30 mg/kg on days 1 and 15) in a 28-day cycle to determine the optimal dose level and schedule and to confirm safety. In parallel, 12 patients were enrolled in the cholangiocarcinoma cohort, in which patients were administered TRK-950 intravenously weekly (10 mg/kg on days 1, 8, 15, and 22) in a 28-day cycle to assess antitumor activity. Patients who successfully completed treatment cycle 1 (28 days) without evidence of significant treatment-related toxicities or clinical evidence of progressive disease continued to receive the treatment. Treatment could continue as long as there was a perceived benefit or until disease progression. The study protocol and other relevant documents were approved by the relevant Institutional Review Boards or ethics committees, and all participants provided written informed consent. This study was conducted in accordance with the Declaration of Helsinki and International Conference on Harmonization Guidelines for Good Clinical Practice.

### Patient eligibility

Patients eligible for the dose escalation cohort were male or female patients ≥18 years of age with histologically confirmed locally advanced or metastatic solid carcinomas who were likely to overexpress CAPRIN-1 and had already received or been considered for all therapies known to confer clinical benefit, with tumor progression after receiving standard/approved chemotherapy or in which there was no approved therapy or that were not amenable to curative treatment. Patients were to have measurable disease per RECIST version 1.1 (primary or metastases), a Karnofsky performance status ≥ 70%, and a life expectancy of at least 3 months. Patients eligible for the colorectal cancer cohort (10 and 30 mg/kg) were histologically confirmed to have locally advanced or metastatic colorectal cancer. The patients eligible for the cholangiocarcinoma cohort (10 mg/kg) had histologically confirmed cholangiocarcinoma. Key exclusion criteria included New York Heart Association Class III or IV; cardiac disease; myocardial infarction within the past 6 months; unstable arrhythmia; evidence of ischemia on ECG; uncontrolled bacterial, viral, or fungal infections; known active infection with human immunodeficiency virus; hepatitis B; hepatitis C; symptomatic brain metastases; serious nonmalignant disease; and treatment with radiotherapy, surgery, chemotherapy, immunotherapy, or investigational therapy within 4 weeks prior to study entry. The representativeness of the study population is included in Supplementary Table S1.

### Assessments

#### Safety, DLTs, and MTD

Safety assessments performed during the study included monitoring of adverse events (AE), physical examination, vital signs, 12-lead electrocardiogram, and laboratory tests (hematology, serum chemistry, and urinalysis). Safety data were collected from the date of written informed consent until 28 days after the administration of the last dose of the study drug. All AEs that occurred after the first dose of the study drug were considered treatment-emergent AEs (TEAE). All AEs were classified using the MedDRA version 19.1 classification system. The severity of toxicity was graded according to the NCI Common Terminology Criteria for Adverse Events version 4.03. A DLT was defined as one of the following toxicities: grade 4 neutropenia lasting ≥5 days, grade 3 or 4 neutropenia with fever and/or infection, grade 4 thrombocytopenia (or grade 3 with bleeding), grade 3 or 4 treatment-related nonhematologic toxicity, and a dosing delay greater than 2 weeks due to TEAEs or related severe laboratory abnormalities. The MTD was the highest dose level that did not meet the toxic dose level definition. The toxic dose level was defined as the lowest dose level at which a DLT was experienced in ≥2 out of a maximum of six patients.

### PK

Serum was collected for PK analysis as described in Supplementary Table S2. The serum concentrations of TRK-950 were measured using a validated immunoassay method. The serum concentrations of TRK-950 from the first dose in cycle 1 were analyzed by noncompartmental analysis using Phoenix WinNonlin (version 8.3, Certara, 2020) to determine the following PK parameters: time to maximum serum concentration, maximum observed serum concentration (C_max_), area under the serum concentration–time curve (AUC) from time zero to the time of the last quantifiable concentration (AUC_last_), AUC extrapolated from time zero to infinity (AUC_inf_), terminal elimination half-life, total body clearance, and apparent steady-state volume of distribution.

Dose proportionality was assessed within the dose escalation cohort using plots of mean C_max_, mean AUC_last_, and mean AUC_inf_ by dose and an empirical model relating the log of the PK parameter linearly to the log of the dose (the “power model”).

### Antitumor activity

Tumor response was determined according to RECIST 1.1 criteria using objective measurements of target lesions, assessment of nontarget lesions, and identification of new lesions. Radiographic tumor burden assessment (CT/MRI) was performed at screening to establish a baseline and was reassessed at the end of every other cycle until cycle 6. After cycle 6 was completed, the tumor burden was assessed every three cycles.

### IHC analysis for CAPRIN-1

CAPRIN-1 expression was examined by the IHC method using a rabbit anti-human CAPRIN-1 monoclonal antibody and was measured for all patients in all cohorts at screening using tumor tissue biopsied from the primary tumor or metastatic lesions at a centralized pathologic laboratory. For the dose escalation cohort, if archival tissue was obtained more than 1 year prior to screening, a new biopsy was required to obtain tissue. For the colorectal cancer and cholangiocarcinoma cohorts, if archival tissue was available from a biopsy taken <18 months prior to screening, it was collected. If archival tissue was obtained >18 months prior to screening, tumor tissue for CAPRIN-1 determination was obtained with a pretreatment biopsy. The proportion of cancer cells that positively stained for CAPRIN-1 on the cell membrane was categorized as follows: no reactivity (0), faint/barely perceptible reactivity (1+), weak to moderate reactivity (2+), and strong reactivity (3+). The category was determined by the intensity of membrane staining and the percentage of stained cells, as shown in Supplementary Table S3.

### Tumor-infiltrating macrophages

Multi-immunofluorescence analysis of tumor-infiltrating macrophages was performed on formalin-fixed, paraffin-embedded tumor tissue samples obtained from each participant in the colorectal cancer and cholangiocarcinoma cohorts. After antigen retrieval (36 minutes at 95°C, pH 8.4), 4-µm-thick sections of tumor specimens obtained from core-needle biopsies at the screening and C1D22 time points were stained with mouse monoclonal anti-human CD68 (IgG2b, clone 298807, R&D Systems) and mouse monoclonal anti-human CD163 (IgG1, clone 10D6, Leica Biosystems) and then incubated with AF647 goat anti-mouse IgG2b and AF488 goat anti-mouse IgG1 (both from Invitrogen). The sections were then counterstained with DAPI (ref D1306, Invitrogen). Macrophage populations, such as CD68^+^/CD163^+^ cells and CD68^+^/CD163^−^ cells, were manually counted on digital images from all available biopsies, and the data were expressed as the number of cells per mm^2^.

### Statistical analysis

The sample size for the dose escalation cohort was based on a 3 + 3 design, whereas that for the other cohorts was determined without statistical considerations. Descriptive statistics were used to evaluate the safety, PK, and other measurements. The 95% confidence intervals for the disease control rate were calculated using the Pearson–Clopper method. Safety analysis was performed on the population that received at least one dose of the study drug. Analysis of antitumor activity was performed on the population that received at least one dose of the study drug and had at least one post-baseline tumor assessment. All analyses were performed using the SAS statistical software (version 9.4; SAS Inc.).

### Data availability

All clinical trial data presented in the article are not publicly available, as they could compromise patient privacy or consent, but are available upon reasonable request by contacting the corresponding author.

## Results

### Patient characteristics

The patient demographics and baseline characteristics are summarized in Table 1, and the CONSORT diagram is shown in Supplementary Fig. S1. The total number of patients enrolled who received at least one dose of the study medication was 36: 10 patients in the dose escalation cohort, 14 patients in the colorectal cancer cohort, and 12 patients in the cholangiocarcinoma cohort. The median age of the patients was 62.5 years (range, 36–78 years). There were a variety of anatomic locations of the individual patients’ primary cancers, with the largest numbers being reported for colorectal cancer and cholangiocarcinoma (15 and 13 patients, respectively). Two patients each had breast, pancreatic, and gastric cancer. Most patients had stage IV disease at the time of study enrollment. All patients had received at least one prior chemotherapy session, and the majority had a history of three or more chemotherapy treatments. Among 14 patients in the colorectal cancer cohort, all were treated with 5-fluorouracil. Additionally, 12 patients received irinotecan, 13 patients received oxaliplatin, 11 patients received bevacizumab, and five patients were treated with an anti-EGFR antibody, including cetuximab. Among 12 patients in the cholangiocarcinoma cohort, all were treated with gemcitabine and cisplatin, and six patients were treated with fluoropyrimidine as a single agent in the adjuvant setting or in combination with irinotecan and/or oxaliplatin. Most patients had either a 3+ or 2+ CAPRIN-1 semiquantitative expression score [15 (41.7%) and 10 (27.8%) patients, respectively].

**Table 1:** Baseline characteristics of treated patients

Characteristics	Dose escalation cohort, 3 mg/kg (*N* = 4)	Dose escalation cohort, 10 mg/kg (*N* = 3)	Dose escalation cohort, 30 mg/kg (*N* = 3)	Colorectal cancer cohort, 10 mg/kg (*N* = 6)	Colorectal cancer cohort, 30 mg/kg (*N* = 8)	Cholangiocarcinoma cohort, 10 mg/kg (*N* = 12)	All patients (*N* = 36)^a^
Age, years [median (min–max)]	63.0 (38, 73)	66.0 (62, 69)	58.0 (47, 72)	65.0 (49, 75)	62.5 (45, 78)	62.5 (36, 78)	62.5 (36, 78)
Sex, *n* (%)							
Male	3 (75.0)	1 (33.3)	0	5 (83.3)	6 (75.0)	5 (41.7)	20 (55.6)
Female	1 (25.0)	2 (66.7)	3 (100.0)	1 (16.7)	2 (25.0)	7 (58.3)	16 (44.4)
Screening KPS category, *n* (%)							
70	0	0	0	1 (16.7)	1 (12.5)	1 (8.3)	3 (8.3)
80	1 (25.0)	1 (33.3)	2 (66.7)	1 (16.7)	1 (12.5)	1 (8.3)	7 (19.4)
90	3 (75.0)	1 (33.3)	0	3 (50.0)	4 (50.0)	7 (58.3)	18 (50.0)
100	0	0	1 (33.3)	1 (16.7)	2 (25.0)	3 (25.0)	7 (19.4)
Anatomic location of primary cancer, *n* (%)							
Breast	1 (25.0)	0	1 (33.3)	0	0	0	2 (5.6)
Colorectal	1 (25.0)	0	0	6 (100.0)	8 (100.0)	0	15 (41.7)
Gastric	2 (50.0)	0	0	0	0	0	2 (5.6)
Pancreatic	0	1 (33.3)	1 (33.3)	0	0	0	2 (5.6)
Cholangiocarcinoma	0	1 (33.3)	0	0	0	12 (100.0)	13 (36.1)
Other^b^	0	1 (33.3)	1 (33.3)	0	0	0	2 (5.6)
Histology, *n* (%)							
Adenocarcinoma	3 (75.0)	3 (100.0)	2 (66.7)	6 (100.0)	7 (87.5)	12 (100.0)	33 (91.7)
Unknown	1 (25.0)	0	0	0	1 (12.5)	0	2 (5.6)
Benign hepatic parenchyma with calcifications	0	0	1 (33.3)	0	0	0	1 (2.8)
Prior systemic therapy							
1 or 2	0	2 (66.7)	0	0	3 (37.5)	10 (83.3)	15 (41.7)
3 or higher	4 (100.0)	1 (33.3)	3 (100.0)	6 (100.0)	5 (62.5)	2 (16.7)	21 (58.3)
Screening CAPRIN-1 score category, *n* (%)							
3+	3 (75.0)	1 (33.3)	2 (66.7)	4 (66.7)	3 (37.5)	2 (16.7)	15 (41.7)
2+	0	1 (33.3)	0	0	3 (37.5)	6 (50.0)	10 (27.8)
1+	0	0	0	0	0	1 (8.3)	1 (2.8)
0	0	0	0	1 (16.7)	0	2 (16.7)	3 (8.3)
Not detected	1 (25.0)	1 (33.3)	1 (33.3)	0	1 (12.5)	1 (8.3)	5 (13.9)

Abbreviation: KPS, Karnofsky performance status.

a Patients who received one or more doses of TRK-950.

b One patient with anal cancer in the dose escalation cohort at 10 mg/kg and the other with appendiceal cancer in the dose escalation cohort at 30 mg/kg.

### Safety

TEAEs reported at CTCAE all grades in >10% or more patients and at CTCAE grade 3 or higher and treatment-related TEAEs are summarized in Table 2. No DLTs were observed during dose escalation, and no MTD was determined in this study. After the administration of TRK-950, all 36 patients (100.0%) had at least one TEAE, and 10 patients (27.8%) had at least one treatment-related TEAE. The TEAEs that the investigators attributed as at least possibly related to the study drug (*n* = 10) were generally mild to moderate, with the exception of one event (2.8%: fatigue) that was considered severe (grade 3) in the cholangiocarcinoma cohort. Fifteen (41.7%) patients had at least one serious adverse event (SAE) out of a total of 21 SAEs. One patient (12.5%) who received 30 mg/kg in the colorectal cancer cohort had an SAE of cerebral thrombosis that was considered to be related to the study drug (grade 2). Notably, in the analysis of patients reporting TEAEs, there was no noticeable pattern either overall or by individual preferred term in the frequency of patients reporting TEAEs during the individual dosing days. For each preferred term, the most commonly reported TEAEs (in ≥20% of patients) were abdominal pain (18 patients, 50.0%), followed by fatigue (14 patients, 38.9%), constipation (11 patients, 30.6%), back pain and nausea (10 patients each, 27.8%), and decreased appetite (nine patients, 25.0%). Treatment-related TEAEs (≥2 patients) included fatigue and nausea (two patients, 5.6%).

**Table 2:** The incidence of AEs

​	All AEs >10%							Grade 3 or higher AEs								
	TRK-950 dose group						​	TRK-950 dose group							​	​
MedDRA preferred term	Dose escalation cohort, 3 mg/kg (*N* = 4)	Dose escalation cohort, 10 mg/kg (*N* = 3)	Dose escalation cohort, 30 mg/kg (*N* = 3)	Colorectal cancer cohort, 10 mg/kg (*N* = 6)	Colorectal cancer cohort, 30 mg/kg (*N* = 8)	Cholangiocarcinoma cohort, 10 mg/kg (*N* = 12)	Total no. of patients (*N* = 36; %)	Drug-related AEs, no. of patients (*N* = 36; %)	Dose escalation cohort, 3 mg/kg (*N* = 4)	Dose escalation cohort, 10 mg/kg (*N* = 3)	Dose escalation cohort, 30 mg/kg (*N* = 3)	Colorectal cancer cohort, 10 mg/kg (*N* = 6)	Colorectal cancer cohort, 30 mg/kg (*N* = 8)	Cholangiocarcinoma cohort, 10 mg/kg (*N* = 12)	Grade ≥3 AEs, total no. of patients (*N* = 36; %)	Drug-related grade ≥3 AEs, no. of patients (*N* = 36; %)
Any TEAE	4	3	3	6	8	12	36 (100.0%)	10 (27.8%)	2	1	3	3	4	7	20 (55.6%)	1 (2.8%)
Abdominal pain	1	2	1	1	5	8	18 (50.0%)	0 (0%)	0	0	1	0	1	3	5 (13.9%)	0 (0%)
Fatigue	1	0	1	1	6	5	14 (38.9%)	2 (5.6%)	0	0	0	0	1	1	2 (5.6%)	1 (2.8%)
Constipation	1	0	2	0	4	4	11 (30.6%)	0 (0%)	0	0	0	0	0	0	0 (0%)	0 (0%)
Back pain	1	0	0	2	4	3	10 (27.8%)	0 (0%)	0	0	0	0	1	1	2 (5.6%)	0 (0%)
Nausea	0	1	0	0	3	6	10 (27.8%)	2 (5.6%)	0	0	0	0	1	0	1 (2.8%)	0 (0%)
Decreased appetite	0	0	0	2	4	3	9 (25.0%)	0 (0%)	0	0	0	0	0	0	0 (0%)	0 (0%)
Pyrexia	1	1	0	0	1	4	7 (19.4%)	0 (0%)	0	0	0	0	0	0	0 (0%)	0 (0%)
Increased blood alkaline phosphatase	0	0	0	2	1	3	6 (16.7%)	0 (0%)	0	0	0	1	1	2	4 (11.1%)	0 (0%)
Diarrhea	0	0	0	2	1	3	6 (16.7%)	0 (0%)	0	0	0	0	0	0	0 (0%)	0 (0%)
Dyspnea	0	0	1	1	1	3	6 (16.7%)	0 (0%)	0	0	1	1	0	0	2 (5.6%)	0 (0%)
Anemia	1	1	1	1	1	0	5 (13.9%)	1 (2.8%)	0	0	0	1	0	0	1 (2.8%)	0 (0%)
Anxiety	0	0	2	1	1	1	5 (13.9%)	0 (0%)	0	0	0	0	0	0	0 (0%)	0 (0%)
Cough	0	1	0	1	1	2	5 (13.9%)	0 (0%)	0	0	0	0	0	0	0 (0%)	0 (0%)
Increased gamma-glutamyltransferase	0	0	0	1	1	3	5 (13.9%)	0 (0%)	0	0	0	1	1	3	5 (13.9%)	0 (0%)
Insomnia	0	0	2	1	0	2	5 (13.9%)	0 (0%)	0	0	0	0	0	0	0 (0%)	0 (0%)
Increased alanine aminotransferase	0	0	0	1	0	3	4 (11.1%)	0 (0%)	0	0	0	0	0	3	3 (8.3%)	0 (0%)
Arthralgia	0	1	0	1	1	1	4 (11.1%)	0 (0%)	0	0	0	1	0	0	1 (2.8%)	0 (0%)
Depression	0	0	1	1	0	2	4 (11.1%)	0 (0%)	0	0	1	0	0	0	1 (2.8%)	0 (0%)
Hypotension	0	1	0	1	0	2	4 (11.1%)	0 (0%)	0	0	0	0	0	0	0(0%)	0 (0%)
Liver disorder	0	0	0	1	1	2	4 (11.1%)	0 (0%)	0	0	0	0	0	0	0 (0%)	0 (0%)
Peripheral edema	1	0	0	2	1	0	4 (11.1%)	0 (0%)	0	0	0	0	0	0	0 (0%)	0 (0%)
Pruritus	1	0	0	0	1	2	4 (11.1%)	1 (2.8%)	0	0	0	0	0	0	0 (0%)	0 (0%)
Rhinorrhea	0	0	0	1	2	1	4 (11.1%)	0 (0%)	0	0	0	0	0	0	0 (0%)	0 (0%)
Somnolence	1	0	1	1	0	1	4 (11.1%)	0 (0%)	0	0	0	0	0	0	0 (0%)	0 (0%)
Vomiting	0	0	0	0	1	3	4 (11.1%)	1 (2.8%)	0	0	0	0	0	0	0 (0%)	0 (0%)

Abdominal pain includes upper abdominal pain and lower abdominal pain.

### Antitumor activity

**Figure 1:**
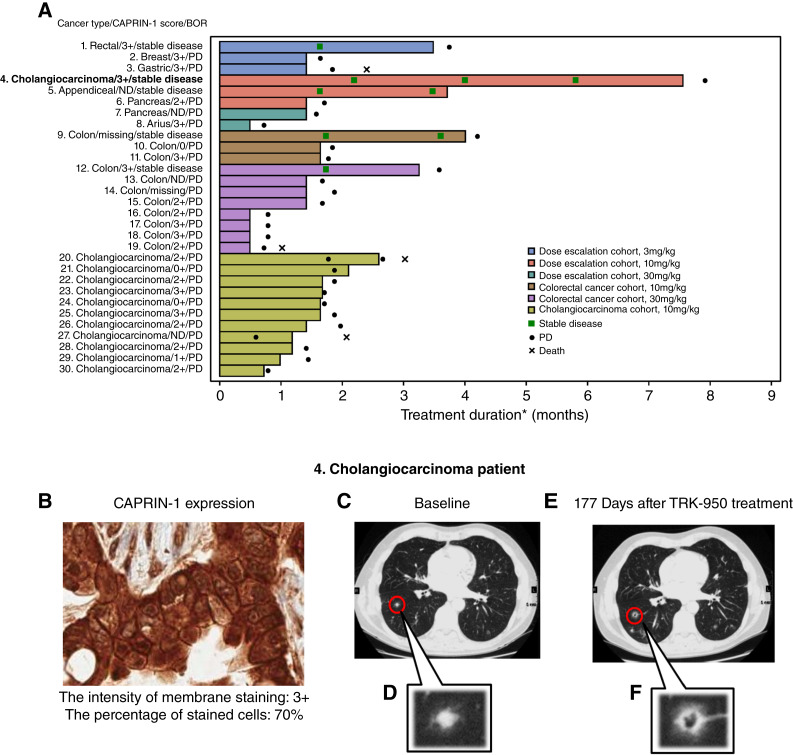
Duration of treatment and response with TRK-950 and representative antitumor activity in patients with cholangiocarcinoma. **A,** Duration of treatment and response with TRK-950. Each bar represents 1 patient. The length of the bar represents the duration of treatment with TRK-950. Treatment duration was defined as follows: (Date of Last Dose – Date of First Dose + 1), and was the period up to the date of the last dose. BOR, best overall response; ND, not detected; PD, progressive disease; missing, no samples to evaluate. **B,** Representative image of CAPRIN-1 expression in patients with cholangiocarcinoma. CAPRIN-1–IHC staining features observed in archival tumor specimens of cholangiocarcinoma. **C–F,** Representative antitumor activity in patients with cholangiocarcinoma. CT scans of the metastatic lung region for a patient receiving TRK-950 10 mg/kg. Images were taken (**C** and **D**) at baseline and (**E** and **F**) 177 days after TRK-950 treatment. **D** and **F** indicate enlarged regions.

Among the 30 patients evaluable for tumor burden assessment in any cohort, five (16.7%) patients had a best overall response of stable disease, including patients with colorectal cancer (three patients), cholangiocarcinoma (one patient), and appendiceal cancer (one patient; Table 3). Of the five patients, three had positive CAPRIN-1 expression (3+). The other two patients could not be adequately evaluated due to missing specimens and lack of tumor cells (Fig. 1A; Supplementary Fig. S2A and S2B). All patients in the cholangiocarcinoma cohort had progressive disease as their best response. However, in the colorectal cancer cohort, two patients had stable disease. One patient had received three prior lines of treatment, including an experimental agent, whereas the other patient had received 12 prior treatments, also including experimental agents. There was no obvious bias in the metastatic sites or tumor burden in these patients; both patients had relatively large sums of target lesions, measuring 360 and 147 mm, respectively. In addition, one patient with cholangiocarcinoma in the dose escalation cohort, treated with 10 mg/kg weekly for 3 weeks followed by a 1-week rest period, remained in the study with stable disease for eight cycles until this patient experienced progression of nontarget lesions in the lung (Fig. 1A). During the treatment period with TRK-950, the tumor marker CA19-9 ranged from 4 to 27 U/mL, and carcinoembryonic antigen ranged from 1.6 to 3.4 ng/mL, with both marker values remaining within the reference range (Supplementary Table S4). In addition, this patient had high intensity (3+) and percentage (70%) of CAPRIN-1 expression (Fig. 1B). Furthermore, several of the pulmonary metastases in CT scans through the four imaging time points showed signs of cavitation, potentially indicative of antitumor activity (Fig. 1C–F).

**Table 3:** Summary of best overall response and disease control rate

No. of patients^a^	Dose escalation cohort, 3 mg/kg (*N* = 3)	Dose escalation cohort, 10 mg/kg (*N* = 3)	Dose escalation cohort, 30 mg/kg (*N* = 2)	Colorectal cancer cohort, 10 mg/kg (*N* = 3)	Colorectal cancer cohort, 30 mg/kg (*N* = 8)	Cholangiocarcinoma cohort, 10 mg/kg (*N* = 11)	All (*N* = 30)
Best overall response categories, *n* (%)							
Complete response	0	0	0	0	0	0	0
Partial response	0	0	0	0	0	0	0
Stable disease	1 (33.3)	2 (66.7)	0	1 (33.3)	1 (12.5)	0	5 (16.7)
Progressive disease	2 (66.7)	1 (33.3)	2 (100.0)	2 (66.7)	7 (87.5)	11 (100.0)	25 (83.3)
Not evaluable	0	0	0	0	0	0	0
Disease control rate^b^:							
*n* (%)	1 (33.3)	2 (66.7)	0	1 (33.3)	1 (12.5)	0	5 (16.7)
95% CI^c^	(0.84–90.57)	(9.43–99.16)	(0.00–84.19)	(0.84–90.57)	(0.32–52.65)	(0.00–28.49)	(5.64–34.72)

Abbreviation: CI, confidence interval.

a *N* represents the number of patients assigned to the respective dose level/cohort and is the denominator for percentages unless otherwise indicated. Response is based on investigator assessment using RECIST 1.1.

b Disease control rate is defined as the proportion of patients who achieved complete response, partial response, or stable disease as their best overall response in accordance with RECIST 1.1 criteria.

c CIs were calculated using the Pearson–Clopper method.

### PK

The PK profile of TRK-950 was assessed from serum concentrations after the first intravenous administration at doses of 3, 10, and 30 mg/kg, administered weekly or biweekly during cycle 1 (Supplementary Fig. S3). The PK parameters based on the noncompartmental analysis are presented in Table 4. The total body clearance, apparent steady-state volume of distribution, and terminal elimination half-life were estimated to be 0.362 mL/hour/kg, 64.1 mL/kg, and 125.5 hours (5.2 days), respectively. C_max_, AUC_last_, and AUC_inf_ increased in a dose-dependent manner, and a dose proportionality analysis with a power model revealed that C_max_ and AUC_last_ were dose-proportional, whereas AUC_inf_ was not (Supplementary Table S5).

**Table 4:** PK parameters of TRK-950

No. of patients	Dose escalation cohort 1, 3 mg/kg (*N* = 4)	Dose escalation cohort 2, 10 mg/kg (*N* = 3)	Dose escalation cohort 3, 30 mg/kg (*N* = 3)	Colorectal cancer cohort, 10 mg/kg (*N* = 6)	Colorectal cancer cohort, 30 mg/kg (*N* = 8)	Cholangiocarcinoma cohort, 10 mg/kg (*N* = 12)	All patients (*N* = 36)
C_max_ (μg/mL)^a^	69.0 (25.4)	203 (12.2)	611 (147)	209 (58)	606 (106)	290 (175)	—
T_max_ (h)^b^	1.6 (1.0, 24.1)	2.0 (1.0, 2.2)	1.5 (1.0, 2.1)	2.0 (1.0, 7.4)	2.1 (1.0, 7.1)	2.1 (1.0, 7.1)	2.0 (1.0, 24.1)
AUC_last_ (h*μg/mL)^a^	5,450 (3,510)	17,500 (3,300)	45,900 (6,900)	15,500 (7,100)	73,200 (18,700)	18,600 (7,600)	—
AUC_inf_ (h*μg/mL)^a^	13,700 (4,200)	29,300 (8,000)	71,900 (8,600)	30,200 (8,900)	96,100 (30,500)	29,500 (12,000)	—
CL (mL/h/kg)^a^	0.230 (0.071)	0.362 (0.109)	0.421 (0.048)	0.362 (0.135)	0.340 (0.103)	0.390 (0.147)	0.362 (0.120)
V_ss_ (mL/kg)^a^	43.7 (9.7)	62.0 (8.1)	69.1 (12.4)	63.1 (18.2)	73.1 (15.1)	60.6 (18.2)	64.1 (16.5)
t_½_ (hours)^b^	133.7 (125.5, 141.9)	128.1 (102.1, 137.9)	115.5 (106.3, 116.5)	106.5 (102.0, 199.6)	162.7 (110.2, 208.8)	108.3 (65.7, 181.1)	125.5 (65.7, 208.8)

Abbreviations: CL, total body clearance; max, maximum; min, minimum; t½, terminal elimination half-life; T_max_, time to maximum serum concentration; V_ss_, apparent steady-state volume of distribution; *N* in the header represents the number of patients assigned to the respective cohort in the PK population and is the denominator for percentages unless otherwise indicated.

a Mean (SD) presented for C_max_, AUC_last_, AUC_inf_, CL, and V_ss_.

b Median (min, max) presented for T_max_ and t_½_.

### Treatment-related changes of tumor-infiltrating macrophages

**Figure 2:**
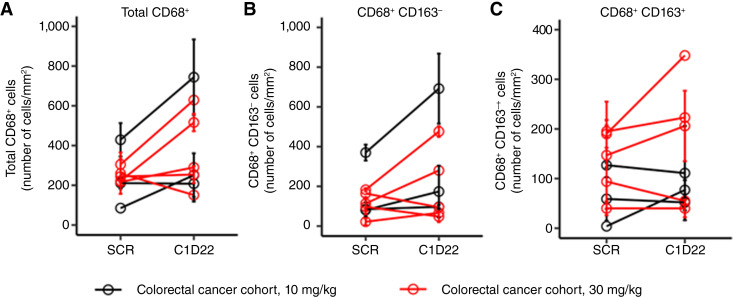
Graphs depicting the density of macrophage subsets pre- and posttreatment with TRK-950. Tumor specimens obtained with core-needle biopsies at the screening and C1D22 time points were subjected to multi-immunofluorescence to detect macrophage infiltrates. Quantitative analyses were performed on digital images of CD68 and CD163 staining. **A,** total CD68^+^ cells; (**B**) CD68^+^/CD163^−^ subsets; (**C**) CD68^+^/CD163^+^ subsets. Error bars indicate SD. C1D22, cycle 1 day 22; SCR, screening.

To characterize the immunologic events associated with the treatment of TRK-950, we evaluated whether TRK-950 promoted an increase in the number of tumor-infiltrating immune cells. In particular, we focused on macrophage infiltration as the primary pharmacologic action of TRK-950, which is considered ADCP, based on the results of nonclinical studies. Matched pretreatment and on-treatment tumor biopsies were available for eight patients in the colorectal cancer cohort and were analyzed by immunofluorescence. All biopsies performed for the analyses were obtained from metastatic sites: the liver in seven cases and the adrenal gland in one case. Analysis of CD68/CD163 staining revealed that the density of macrophages (total CD68^+^ cells) significantly increased after treatment in four patients, decreased in one patient, and remained stable in the other patients (Fig. 2A). This increase was mostly in CD163^−^ macrophages (M1) in two patients and both CD163^−^ and CD163^+^ subsets in the other patients (Fig. 2B and C).

## Discussion

In this phase I study, TRK-950 monotherapy demonstrated a favorable safety profile up to the maximum dose of 30 mg/kg weekly for 3 weeks, followed by a 1-week rest period, and was well tolerated in the patient population with advanced/metastatic solid tumors. In addition, no DLTs were observed, and the MTD was not reached. There was no significant dose dependence for the frequency or severity of TEAEs. Furthermore, the most common AEs reported were abdominal pain, fatigue, constipation, back pain, nausea, and decreased appetite, mainly grade 1/2 in severity, and no particular pattern was observed in the frequency of TEAEs reported across different dose levels among patients who reported TEAEs. These favorable safety profile data suggest that AEs are not derived from specific organ or cell characteristics and are consistent with the safety profile of TRK-950 in cynomolgus monkeys. Although CAPRIN-1 expression was observed at a low level in the cytoplasm of some normal tissues, CAPRIN-1 was barely expressed on the cell membranes of many normal tissues (20). This notable characteristic suggests that the safety profile shown in this study is consistent with the lack of expression of the binding site for TRK-950 in many normal tissues.

The PK profile of TRK-950 is similar to that of other IgG1 therapeutic antibodies (21–23). The distribution volume of TRK-950 was approximately 1.5 times the total plasma volume in humans (3 L for a 70 kg body weight; ref. 24). It has been suggested that TRK-950, which shows high affinity for CAPRIN-1, a protein strongly expressed on the surface of cancer cells, is mainly distributed in the circulating blood and tumor tissue. In the dose range of 3 to 30 mg/kg and serum concentration range of approximately 20 to 850 μg/mL, no steep decline in serum concentration was observed, indicating that a nonlinear PK profile due to target-mediated drug disposition clearance was not present (25, 26). This suggests that TRK-950 fully saturated its binding to CAPRIN-1 within this dose range.

Although no objective responses were observed in the dose escalation cohort, the best response of stable disease was observed in two cases of colorectal cancer and one case of cholangiocarcinoma. Among these three cases, CAPRIN-1 expression could not be evaluated in one case, but the remaining two cases exhibited high expression (3+). We previously demonstrated that the level of CAPRIN-1 expression was crucial for the antitumor effect of TRK-950 (20). Therefore, we selected these two cancer types, which showed the best response, to explore the correlation between TRK-950 efficacy and CAPRIN-1 expression, as well as the characterization of the immunologic events associated with the treatment of TRK-950.

One of the objectives of this study was to establish a recommended dose of TRK-950 for future clinical trials. Because no DLTs were observed at dose levels up to 30 mg/kg and the MTD was not reached, dose-related safety concerns could not be factored into the dose-finding estimates. Additionally, the safety and tolerability of TRK-950 monotherapy at doses ranging from 3 to 30 mg/kg were favorable, with dose-proportional exposure. The PK profile suggested complete binding with CAPRIN-1 at 3 to 30 mg/kg, and the dose–exposure relationship also demonstrated proportionality. Thus, the dose range of 3 to 30 mg/kg was supported as the recommended dose of TRK-950 for future clinical trials. Furthermore, PK/pharmacodynamic analysis (unpublished data) utilizing nonclinical data (20) and the PK profile of this study revealed that the minimally active dose and saturated active dose were determined to be 10 mg/kg, which led to the selection of 10 mg/kg.

Although the assessment of the antitumor activity of TRK-950 was not the primary objective of this first-in-human study and the enrolled patients were heavily pretreated, disease control was achieved in five patients, including one patient with cholangiocarcinoma who was treated for approximately 8 months. Interestingly, 70% of the tumor cells in this patient were identified as CAPRIN-1 at a strong intensity (3+). Owing to the limited number of patients, no clear conclusions could be drawn about the association between CAPRIN-1 expression and the antitumor activity of TRK-950. Further investigation into these relationships is required.

In our previous nonclinical studies, we demonstrated that the mechanism of action of TRK-950 involved ADCP and antibody-dependent cell-mediated cytotoxicity activity. In addition, we analyzed tumor-infiltrating immune cells in a mouse xenograft model when TRK-950 was administered and found that macrophages, which are important for ADCP activity, were the main infiltrating cells (20). In this study, we analyzed changes in tumor-infiltrating immune cells in the tumor tissues of patients before and after TRK-950 administration. Although this analysis was performed on a small subset of patients with colorectal cancer, we observed that the number of macrophages was clearly increased after TRK-950 treatment in four of eight patients with colorectal cancer. This increase was mostly associated with CD163^−^ macrophages (M1) in two patients and both CD163^−^ and CD163^+^ subsets in two patients. Although it should be noted that these patients did not respond to TRK-950 treatment, the results support the data obtained in nonclinical studies, as they suggest that TRK-950 treatment may increase macrophage infiltration into the tumor in some patients.

The favorable safety profile of TRK-950 suggests that it may be a suitable candidate for radiopharmaceutical development and may be safely used in combination with a standard treatment that has strong cytotoxicity and activates immune cells. Cytotoxic drugs such as cisplatin and doxorubicin have been reported to have a direct activating effect on antigen-presenting cells (27). In addition, bevacizumab (an IgG1 antibody targeting VEGF-A) has been shown to normalize the vascular system and promote the maturation of antigen-presenting cells (28). Furthermore, we showed that TRK-950 had synergistic antitumor effects in combination with chemotherapeutics, including gemcitabine, cisplatin, and the monoclonal antibody bevacizumab, suggesting that the combination of TRK-950 with standard therapeutic agents that have a direct activating effect on antigen-presenting cells and standard therapeutic agents with an inhibitory effect on angiogenesis would be expected to have a synergistic effect on each other (unpublished data).

In conclusion, this study showed that TRK-950 has a favorable safety profile and can be safely administered to patients with advanced solid tumors. In addition, preliminary evidence of potential antitumor activity in patients with high CAPRIN-1 expression in tumors was observed, thus providing initial clinical experience and a rationale for the further development of TRK-950 in patients with advanced solid tumors expressing CAPRIN-1. TRK-950 is currently in a Ph-Ib study in combination with standard-of-care treatments (chemotherapy, immune checkpoint inhibitors, and targeted agents) for various cancers, including advanced gastric, ovarian, and renal cancer (NCT03872947), and a phase II clinical study in patients with advanced gastric cancer (NCT06038578).

## Supplementary Material

Figure S1 diagram

Table S1 Representativeness of Study Participants

Figure S2 water fall

Table S2 Summary of the blood sampling schedule

Figure S3 Pharmacokinetic profile

Table S3 The criteria for CAPRIN-1 Expression Assessment

Table S4 Tumor Marker Values

Table S5 Power model analysis for dose proportionality relationship

## References

[1] Grill B , Wilson GM, Zhang K-X, Wang B, Doyonnas R, Quadroni M, . Activation/division of lymphocytes results in increased levels of cytoplasmic activation/proliferation-associated protein-1: prototype of a new family of proteins. J Immunol 2004;172:2389–400. 14764709 10.4049/jimmunol.172.4.2389

[2] Wang B , David MD, Schrader JW. Absence of caprin-1 results in defects in cellular proliferation. J Immunol 2005;175:4274–82. 16177067 10.4049/jimmunol.175.7.4274

[3] Bidet K , Dadlani D, Garcia-Blanco MA. G3BP1, G3BP2 and CAPRIN1 are required for translation of interferon stimulated mRNAs and are targeted by a dengue virus non-coding RNA. PLoS Pathog 2014;10:e1004242. 24992036 10.1371/journal.ppat.1004242 PMC4081823

[4] El Fatimy R , Tremblay S, Dury AY, Solomon S, De Koninck P, Schrader JW, . Fragile X mental retardation protein interacts with the RNA-binding protein Caprin1 in neuronal RiboNucleoProtein complexes [corrected]. PLoS One 2012;7:e39338. 22737234 10.1371/journal.pone.0039338 PMC3380850

[5] Hou S , Kumar A, Xu Z, Airo AM, Stryapunina I, Wong CP, . Zika virus hijacks stress granule proteins and modulates the host stress response. J Virol 2017;91:e00474–17. 28592527 10.1128/JVI.00474-17 PMC5533921

[6] Kolobova E , Efimov A, Kaverina I, Rishi AK, Schrader JW, Ham AJ, . Microtubule-dependent association of AKAP350A and CCAR1 with RNA stress granules. Exp Cell Res 2009;315:542–55. 19073175 10.1016/j.yexcr.2008.11.011 PMC2788823

[7] Martin S , Bellora N, González-Vallinas J, Irimia M, Chebli K, de Toledo M, . Preferential binding of a stable G3BP ribonucleoprotein complex to intron-retaining transcripts in mouse brain and modulation of their expression in the cerebellum. J Neurochem 2016;139:349–68. 27513819 10.1111/jnc.13768

[8] Nakayama K , Ohashi R, Shinoda Y, Yamazaki M, Abe M, Fujikawa A, . RNG105/caprin1, an RNA granule protein for dendritic mRNA localization, is essential for long-term memory formation. Elife 2017;6:e29677. 29157358 10.7554/eLife.29677 PMC5697933

[9] Shiina N , Yamaguchi K, Tokunaga M. RNG105 deficiency impairs the dendritic localization of mRNAs for Na+/K+ ATPase subunit isoforms and leads to the degeneration of neuronal networks. J Neurosci 2010;30:12816–30. 20861386 10.1523/JNEUROSCI.6386-09.2010 PMC6633578

[10] Solomon S , Xu Y, Wang B, David MD, Schubert P, Kennedy D, . Distinct structural features of caprin-1 mediate its interaction with G3BP-1 and its induction of phosphorylation of eukaryotic translation initiation factor 2alpha, entry to cytoplasmic stress granules, and selective interaction with a subset of mRNAs. Mol Cell Biol 2007;27:2324–42. 17210633 10.1128/MCB.02300-06 PMC1820512

[11] Wu Y , Zhu J, Huang X, Du Z. Crystal structure of a dimerization domain of human Caprin-1: insights into the assembly of an evolutionarily conserved ribonucleoprotein complex consisting of Caprin-1, FMRP and G3BP1. Acta Crystallogr D Struct Biol 2016;72:718–27. 27303792 10.1107/S2059798316004903 PMC4908866

[12] Gong B , Hu H, Chen J, Cao S, Yu J, Xue J, . Caprin-1 is a novel microRNA-223 target for regulating the proliferation and invasion of human breast cancer cells. Biomed Pharmacother 2013;67:629–36. 23953883 10.1016/j.biopha.2013.06.006

[13] Guo X-M , Zhu F-F, Pan L-W, Chen J-L, Lai J-C, Wu H-X, . Caprin-1 promotes HepG2 cell proliferation, invasion and migration and is associated with poor prognosis in patients with liver cancer. Oncol Lett 2020;20:1761–71. 32724419 10.3892/ol.2020.11712 PMC7377179

[14] Qiu Y-Q , Yang C-W, Lee Y-Z, Yang R-B, Lee C-H, Hsu H-Y, . Targeting a ribonucleoprotein complex containing the caprin-1 protein and the c-Myc mRNA suppresses tumor growth in mice: an identification of a novel oncotarget. Oncotarget 2015;6:2148–63. 25669982 10.18632/oncotarget.3236 PMC4385842

[15] Sabile AA , Arlt MJ, Muff R, Husmann K, Hess D, Bertz J, . Caprin-1, a novel Cyr61-interacting protein, promotes osteosarcoma tumor growth and lung metastasis in mice. Biochim Biophys Acta 2013;1832:1173–82. 23528710 10.1016/j.bbadis.2013.03.014

[16] Shi Q , Zhu Y, Ma J, Chang K, Ding D, Bai Y, . Prostate cancer-associated SPOP mutations enhance cancer cell survival and docetaxel resistance by upregulating Caprin1-dependent stress granule assembly. Mol Cancer 2019;18:170. 31771591 10.1186/s12943-019-1096-x PMC6878651

[17] Tan N , Dai L, Liu X, Pan G, Chen H, Huang J, . Upregulation of caprin1 expression is associated with poor prognosis in hepatocellular carcinoma. Pathol Res Pract 2017;213:1563–7. 29037839 10.1016/j.prp.2017.07.014

[18] Teng Y , Ren Y, Hu X, Mu J, Samykutty A, Zhuang X, . MVP-mediated exosomal sorting of miR-193a promotes colon cancer progression. Nat Commun 2017;8:14448. 28211508 10.1038/ncomms14448 PMC5321731

[19] Yang Z-S , Qing H, Gui H, Luo J, Dai L-J, Wang B. Role of caprin-1 in carcinogenesis. Oncol Lett 2019;18:15–21. 31289466 10.3892/ol.2019.10295 PMC6540157

[20] Okano F , Saito T, Minamida Y, Kobayashi S, Ido T, Miyauchi Y, . Identification of membrane-expressed CAPRIN-1 as a novel and universal cancer target, and generation of a therapeutic anti-CAPRIN-1 antibody TRK-950. Cancer Res Commun 2023;3:640–58. 37082579 10.1158/2767-9764.CRC-22-0310 PMC10112292

[21] Keizer RJ , Huitema ADR, Schellens JHM, Beijnen JH. Clinical pharmacokinetics of therapeutic monoclonal antibodies. Clin Pharmacokinet 2010;49:493–507. 20608753 10.2165/11531280-000000000-00000

[22] Ovacik M , Lin K. Tutorial on monoclonal antibody pharmacokinetics and its considerations in early development. Clin Transl Sci 2018;11:540–52. 29877608 10.1111/cts.12567 PMC6226118

[23] Ryman JT , Meibohm B. Pharmacokinetics of monoclonal antibodies. CPT Pharmacometrics Syst Pharmacol 2017;6:576–88. 28653357 10.1002/psp4.12224 PMC5613179

[24] Davies B , Morris T. Physiological parameters in laboratory animals and humans. Pharm Res 1993;10:1093–5. 8378254 10.1023/a:1018943613122

[25] An G . Concept of pharmacologic target-mediated drug disposition in large-molecule and small-molecule compounds. J Clin Pharmacol 2020;60:149–63. 31793004 10.1002/jcph.1545 PMC7472685

[26] Krippendorff B-F , Kuester K, Kloft C, Huisinga W. Nonlinear pharmacokinetics of therapeutic proteins resulting from receptor mediated endocytosis. J Pharmacokinet Pharmacodyn 2009;36:239–60. 19554432 10.1007/s10928-009-9120-1 PMC2718226

[27] Bracci L , Schiavoni G, Sistigu A, Belardelli F. Immune-based mechanisms of cytotoxic chemotherapy: implications for the design of novel and rationale-based combined treatments against cancer. Cell Death Differ 2014;21:15–25. 23787994 10.1038/cdd.2013.67 PMC3857622

[28] Rahma OE , Hodi FS. The intersection between tumor angiogenesis and immune suppression. Clin Cancer Res 2019;25:5449–57. 30944124 10.1158/1078-0432.CCR-18-1543

