# Supplementary figures and images for: Phase I First-in-Human Study of TRK-950, an IgG1 Antibody Specific to CAPRIN-1, in Patients with Advanced Solid Tumors
Source: Cancer Res Commun. 2025 Jul 11;5(7):1119–28. doi: 10.1158/2767-9764.CRC-25-0123 (PMC12246539; doi:10.1158/2767-9764.CRC-25-0123)

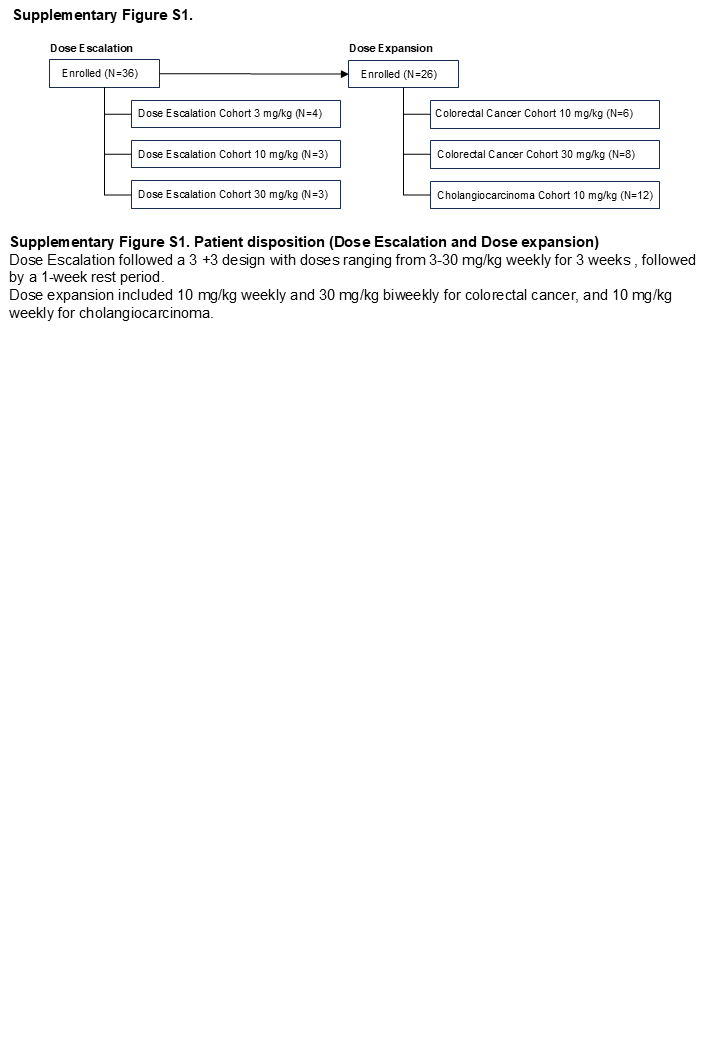

Supplement: Figure S1 — diagram [file crc-25-0123_figure_s1_suppsf1.png]

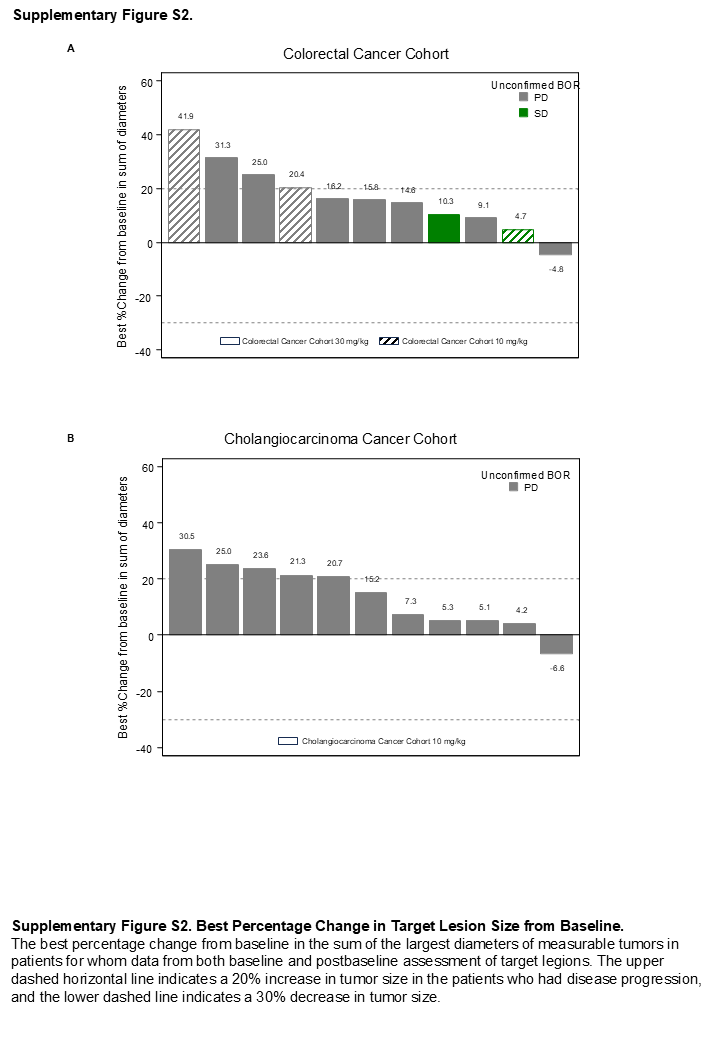

Supplement: Figure S2 — water fall [file crc-25-0123_figure_s2_suppsf2.png]

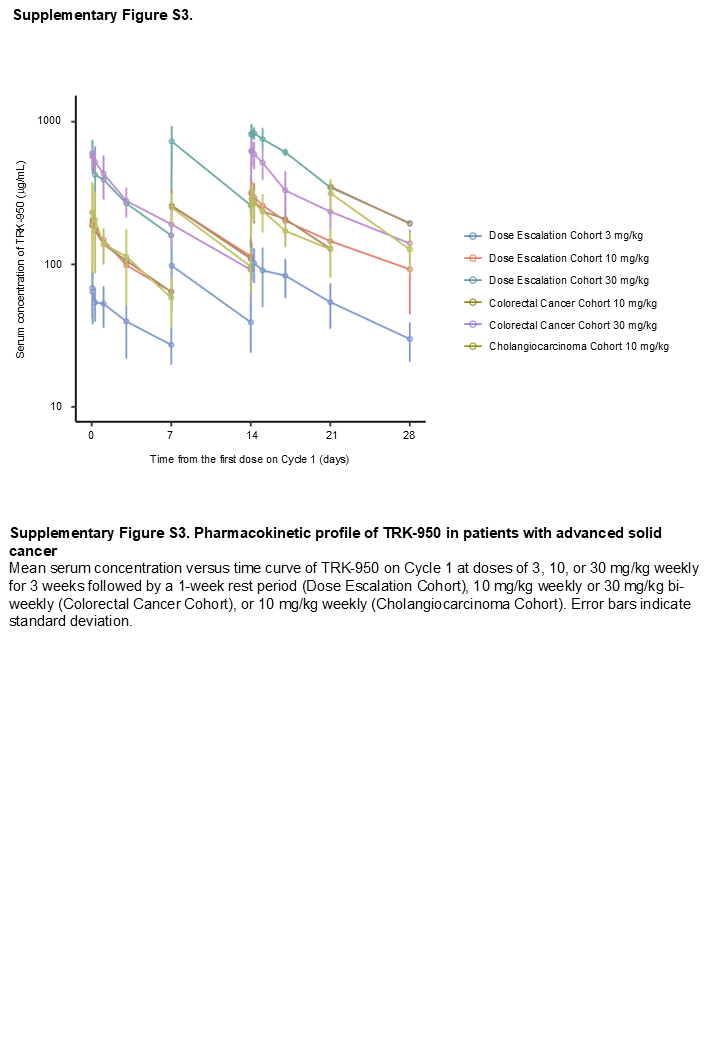

Supplement: Figure S3 — Pharmacokinetic profile [file crc-25-0123_figure_s3_suppsf3.png]
