# Supplementary material for: Phase I First-in-Human Study of TRK-950, an IgG1 Antibody Specific to CAPRIN-1, in Patients with Advanced Solid Tumors
Source: Cancer Res Commun. 2025 Jul 11;5(7):1119–28. doi: 10.1158/2767-9764.CRC-25-0123 (PMC12246539; doi:10.1158/2767-9764.CRC-25-0123)
Supplement: Table S1 — Representativeness of Study Participants [file crc-25-0123_table_s1_suppst1.pdf]

Supplementary Table S1. Representativeness of Study Participants

**Cancer type(s)/subtype(s)/stage(s)/condition Considerations related to:**	**Advanced solid tumors**
Sex	Cancer is among the leading causes of death in the worldwide including United States (US) and European Union (EU). The incidence rate for all cancers combined was slightly higher in men (212.5 per 100,000) than in women (186.2 per 100,000) in 2022. In men, the three cancer sites with the highest age standardized rates (ASRs) in descending order were lung, prostate, and colorectal cancer (40.1, 35.5, and 27.3 per 100,000, respectively) in higher Human Development Index countries and prostate, lung, and lip and oral cavity cancer (12.6, 10.5, and 10.0 per 100,000, respectively) in lower Human Development Index countries. In women, incidence rates for breast cancer far exceed those of other cancers in both transitioned (54.1 per 100,000) and transitioning (30.8 per 100,000) countries, followed by lung cancer (20.7 per 100,000) in transitioned countries and cervical cancer (19.3 per 100,000)in transitioning countries[1].
Age	Cancer is a major societal, public health, and economic problem in the 21st century, responsible for almost one in six deaths (16.8%) and one in four deaths (22.8%) from noncommunicable diseases (NCDs) worldwide. The disease causes three in 10 global premature deaths from NCDs (30.3% in those aged 30–69 years), and it is among the three leading causes of death in this age group in 177 of 183 countries[1]. In the US the median age of any types of cancer diagnosis is 66 years[3] and median age at diagnosis of the most common solid tumors follows a similar pattern[3]. New 2022 estimates for EU Member States show that 31% of men and 25% of women are expected to be diagnosed with cancer before reaching the age of 75 years. 14% of men and 9% of women are estimated to die from cancer before reaching 75 years[4].
Race/ethnicity	Overall cancer incidence is highest among AIAN people(478.8), followed closely by Whit (474.3) and Black people (459.7) in the US although sex specific incidence is highest in Black men, among whom rates during 2016–2020 were 79% higher than those in Asian American or Pacific Islander (AAPI) men (533.9 vs. 299 per 100,000), who have the lowest rates of any sex race group[2].
Geography	Almost one half of all cases (49.2%) and the majority (56.1%) of cancer deaths globally were estimated to occur in Asia in 2022, where 59.2% of the world's population resides (Figure 1B). The cancer mortality burden in the African and Asian regions is disproportionately greater than the corresponding incidence burden. This reflects the respective distribution of cancer types alongside comparatively higher case fatality rates on these continents in part because of late stage diagnoses. Europe has a disproportionately higher cancer incidence and mortality burden, given that the continent has one fifth of the global cancer cases (22.4%) and cancer deaths (20.4%) yet less than 10% of the global population (9.6%)[1].
Other considerations	In the United States 181 rare cancers were identified with overall incidence rates of fewer than 6 cases per 100,000 per year during 2009 through 2013. Together, these rare cancers represent 20% of all cancers diagnosed in the United States during 2009 through 2013[5]. In Europe, rare cancers (those with incidence rates of < 6 per 100 000 people per year) represent a substantial burden of disease. About 5.1 million people in the European Union and the United Kingdom are affected by rare cancers, and more than 650 000 new cases are diagnosed per year. Although individually each of the 198 currently identified rare cancers is considered rare, collectively they account for about 24% of all cancer cases diagnosed each year[6].
Overall representativeness of this study	This study was a relatively small study due to the limit of the number of enrolled patient but included patients with a wide range of tumor types. 36 participants were enrolled in this study and presented with 7 different solid tumors (15 colorectal, 13 cholangiocarcinoma, 2 each of breast, gastric, pancreatic, and 1 each of appendiceal and Anal).<br><br>The median age of participants was 62.5 years (range 36–78), and there was a roughly equal proportion of males (n = 20; 55.6%) and females (n = 16; 44.4%), consistent with global cancer incidence. Among the 15 patients for whom racial information was obtained, most patients were White (n=14; 93.3%), which does not reflect the more diverse racial/ethnic backgrounds of the populations in the US and France where the patients were enrolled in two states/cities.<br><br>In this study, appendiceal cancer was registered as a rare cancer case, but the expression of CAPRIN-1, the target of TRK-950, was not detected (ND). However, as previously reported, CAPRIN-1 is expressed in some types of rare cancers as well. Therefore, rare cancers could be considered potential targets for treatment, similar to more common cancers.

Supplementary Table S1. Representativeness of Study Participants (continued)

1. Bray F, Laversanne M, Sung H, Ferlay J, Siegel RL, Soerjomataram I, et al. Global cancer statistics 2022: GLOBOCAN estimates of incidence and mortality worldwide for 36 cancers in 185 countries. CA Cancer J Clin 2024;74(3):229-63 doi 10.3322/caac.21834.
2. Siegel RL, Wagle NS, Cercek A, Smith RA, Jemal A. Colorectal cancer statistics, 2023. CA Cancer J Clin 2023;73(3):233-54 doi 10.3322/caac.21772.
3. National Cancer Institute: Surveillance, Epidemiology and End Results Program. Age and Cancer Risk. https://www.cancer.gov/about-cancer/causes-prevention/risk/age. Updated March 5, 2021. Accessed February 13, 2025
4. European Cancer Information System. Cancer Factsheets in EU-27 countries - 2022 https://ecis.jrc.ec.europa.eu/cancer-factsheets-eu-27-countries-2022
5. DeSantis CE, Kramer JL, Jemal A. The burden of rare cancers in the United States. CA Cancer J Clin 2017;67(4):261-72 doi 10.3322/caac.21400.
6. Gatta G, van der Zwan JM, Casali PG, Siesling S, Dei Tos AP, Kunkler I, et al. Rare cancers are not so rare: the rare cancer burden in Europe. Eur J Cancer 2011;47(17):2493-511 doi 10.1016/j.ejca.2011.08.008.
7. Okano F, Saito T, Minamida Y, Kobayashi S, Ido T, Miyauchi Y, et al. Identification of Membrane-expressed CAPRIN-1 as a Novel and Universal Cancer Target, and Generation of a Therapeutic Anti-CAPRIN-1 Antibody TRK-950. Cancer Res Commun 2023;3(4):640-58 doi 10.1158/2767-9764.CRC-22-0310.
