# Supplementary material for: Phase I First-in-Human Study of TRK-950, an IgG1 Antibody Specific to CAPRIN-1, in Patients with Advanced Solid Tumors
Source: Cancer Res Commun. 2025 Jul 11;5(7):1119–28. doi: 10.1158/2767-9764.CRC-25-0123 (PMC12246539; doi:10.1158/2767-9764.CRC-25-0123)
Supplement: Table S2 — Summary of the blood sampling schedule [file crc-25-0123_table_s2_suppst2.pdf]

Supplementary Table S2. Summary of the blood sampling schedule for the analysis of TRK-950 concentration

Study Protocol Number	Cohort	Dosage	Number of Patients	PK Sampling Schedule
950P1V01	Dose-escalation Cohort	3, 10 and 30 mg/kg QW, i.v. over 60 min.<br>Day 1, 8 and 15 for 28 days cycle.	4<br>(3 mg/kg)<br><br>3<br>(10 mg/kg)<br><br>3<br>(30 mg/kg)	Cycle 1,<br>Day 1; Pre-dose, 5 min, 1, 6, 24 and 72 h after end of infusion (EOI)<br>Day 8; Pre-dose and 5 min after EOI<br>Day 15; Pre-dose, 5 min, 1, 6, 24 and 72 h after EOI<br>Day 22; During visit<br><br>Cycle 2 and beyond,<br>Day 1, 8 and 15; Pre-dose and 5 min after EOI<br>Follow up visit
	Colorectal Cancer Cohort	10 mg/kg QW, i.v. over 60 min.<br>Day 1, 8, 15 and 22 for 28 days cycle.	8	Cycle 1,<br>Day 1; Pre-dose, 5 min, 1, 6, 24 and 72 h after end of infusion (EOI)<br>Day 8; Pre-dose and 5 min after EOI<br>Day 15; Pre-dose, 5 min, 1, 6, 24 and 72 h after EOI<br>Day 22; Pre-dose and 5 min after EOI<br><br>Cycle 2 and beyond,<br>Day 1, 8, 15 and 22; Pre-dose and 5 min after EOI<br>Follow up visit
		30 mg/kg Q2W, , i.v. over 60 min.<br>Day 1 and 15 for 28 days cycle.	8	Cycle 1,<br>Day 1; Pre-dose, 5 min, 1, 6, 24 and 72 h after end of infusion (EOI)<br>Day 8; During visit<br>Day 15; Pre-dose, 5 min, 1, 6, 24 and 72 h after EOI<br>Day 22; During visit<br><br>Cycle 2 and beyond,<br>Day 1 and 15; Pre-dose and 5 min after EOI<br>Follow up visit
	Cholangiocarcinoma Cohort	10 mg/kg QW, i.v. over 60 min.<br>Day 1, 8, 15 and 22 for 28 days cycle.	12	Cycle 1,<br>Day 1; Pre-dose, 5 min, 1, 6, 24 and 72 h after end of infusion (EOI)<br>Day 8; Pre-dose and 5 min after EOI<br>Day 15; Pre-dose, 5 min, 1, 6, 24 and 72 h after EOI<br>Day 22; Pre-dose and 5 min after EOI<br><br>Cycle 2 and beyond,<br>Day 1, 8, 15 and 22; Pre-dose and 5 min after EOI<br>Follow up visit
