# Supplementary material for: Phase I First-in-Human Study of TRK-950, an IgG1 Antibody Specific to CAPRIN-1, in Patients with Advanced Solid Tumors
Source: Cancer Res Commun. 2025 Jul 11;5(7):1119–28. doi: 10.1158/2767-9764.CRC-25-0123 (PMC12246539; doi:10.1158/2767-9764.CRC-25-0123)
Supplement: Table S3 — The criteria for CAPRIN-1 Expression Assessment [file crc-25-0123_table_s3_suppst3.pdf]

Supplementary Table S3. The criteria for CAPRIN-1 Expression Assessment

Scoring	CAPRIN-1 expression assessment	Intensity score	Membrane staining percent of tumor cells
0	Negative	0<br>Negative	-
		1+ to 3+<br>membranous staining	complete or incomplete membrane staining < 10% of tumor cells.
1+	Negative	1+<br>faint/barely perceptible membranous staining	complete or incomplete membrane staining >= 10% of tumor cells.
2+	Positive	2+<br>Weak to moderate membranous staining	complete or incomplete membrane staining >= 10% of tumor cells.
3+	Positive	3+<br>Strong membranous staining	complete or incomplete membrane staining >= 10% of tumor cells.

Decide the scoring based on intensity and count of majority in the case that both majority and minority on which each score is different are in a cancer tissue.
Count only complete or incomplete membrane staining tumor cells. Do not score cytoplasmic staining in tumor cells as positive staining.
Do not count non-tumor cells such as endothelial cells, fibroblasts or immune cells.
