# Supplementary material for: Phase I First-in-Human Study of TRK-950, an IgG1 Antibody Specific to CAPRIN-1, in Patients with Advanced Solid Tumors
Source: Cancer Res Commun. 2025 Jul 11;5(7):1119–28. doi: 10.1158/2767-9764.CRC-25-0123 (PMC12246539; doi:10.1158/2767-9764.CRC-25-0123)
Supplement: Table S4 — Tumor Marker Values [file crc-25-0123_table_s4_suppst4.pdf]

Supplementary Table S4. Range of Tumor Marker Values In Patients with Cholangiocarcinoma

Tumor marker	Minimum	Maximum
CEA (ng/ml)	1.6	3.4
CA-19-9 (U/ml)	4.0	27
