# Supplementary material for: Phase I First-in-Human Study of TRK-950, an IgG1 Antibody Specific to CAPRIN-1, in Patients with Advanced Solid Tumors
Source: Cancer Res Commun. 2025 Jul 11;5(7):1119–28. doi: 10.1158/2767-9764.CRC-25-0123 (PMC12246539; doi:10.1158/2767-9764.CRC-25-0123)
Supplement: Table S5 — Power model analysis for dose proportionality relationship [file crc-25-0123_table_s5_suppst5.pdf]

## Supplementary Table S5. Power model analysis for dose proportionality relationship

| Parameter | Estimate of slope | 90% confidential interval |
|---|---|---|
| $C_{max}$ | 0.956 | 0.800, 1.113 |
| $AUC_{last}$ | 1.060 | 0.657, 1.463 |
| $AUC_{inf}$ | 0.735 | 0.558, 0.913 |

$AUC_{inf}$ = area under the serum concentration-time curve (AUC) extrapolated to infinity; $AUC_{last}$ = AUC from time zero to the time of the last quantifiable concentration; $C_{max}$ = maximum serum concentration.
